# CLIMATE CHANGE: Health Scenarios for a Warming World

**DOI:** 10.1289/ehp.118-a382

**Published:** 2010-09

**Authors:** Catherine M. Cooney

**Affiliations:** **Catherine M. Cooney**, a science writer in Washington, DC, has written for *Environmental Science & Technology* and Greenwire

A large fraction of emissions of carbon dioxide (CO_2_)—the greenhouse gas produced by human activities in the greatest quantities—is long lived in the atmosphere, so decisions made today to continue adding CO_2_ into the atmosphere may lock future generations into a range of human health and environmental impacts, some of which could become very severe, according to a committee of the National Research Council (NRC). In a report that looks at the short- and long-term effects of the stabilization of Earth’s temperature, the NRC committee quantifies, as much as possible, the outcomes of different stabilization targets for the planet, with a focus on the United States.^1^

The report synthesizes global warming science in myriad fields along with research on the potential impacts for human health and other arenas. Then the committee adds a twist: rather than expressing climate goals in terms of stabilizing atmospheric concentrations of CO_2_, the authors assess such goals using global mean temperature change as the primary metric. The twist allows the authors to link the potential impacts from climate change more directly to temperature change.

Research to date suggests many potential impacts can be directly linked to temperature, or to things that can be themselves linked to temperature (e.g., precipitation), although some (e.g., ocean acidification) are linked directly to CO_2_ concentration, says Damon Matthews of Concordia University, a report coauthor. “But in this report we were . . . noting the additional impacts you would expect for a given degree in global temperature change,” says Katharine Hayhoe of Texas Tech University, another coauthor. Given the anticipated impacts for anywhere from a 1- to 5-°C global temperature increase, the panel “worked backwards and said, ‘If we picked a temperature target based on a risk that is acceptable [to society], then what does that imply regarding the CO_2_ levels we must aim for?’” according to Hayhoe.

Things shifted for climate change researchers about five years ago, when climate models began to factor in the carbon cycle, making it easier to include specific CO_2_ emissions scenarios and link them to temperature, says Matthews. In 2009 Matthews and colleagues described the framework for linking the temperature response to carbon emissions, a construct known as the carbon climate response.^2^ The carbon climate response—the ratio of temperature change to cumulative carbon emissions—“allows CO_2_-induced global mean temperature change to be inferred directly from cumulative carbon emissions,” Matthews et al. wrote.^2^ Three other papers published the same year^3^–^5^ proposed a similar framework and demonstrated “a remarkably consistent temperature response to a given level of cumulative carbon emissions,” the NRC report notes.^1^

The NRC report discusses three main types of health-related stress expected from rising average temperatures: illness and infectious diseases carried by animal hosts and mosquitoes and other vectors, heat-related illness and deaths, and health problems due to air pollution (e.g., related to increased ozone formation) and water contamination (e.g., related to more frequent heavy downpours).

In one discussion, the report summarizes research on a 1995 Chicago heat wave that resulted in 692 heat-related deaths within the city^6^ and extrapolates to predict how many heat waves and deaths might occur with each degree of temperature rise. For instance, under a 2°C change in global mean temperature, annual average mortality rates are projected to equal those of 1995, whereas under a 4°C change in global mean temperature, annual average mortality is projected to be twice 1995 levels, and 1995-like heat waves are predicted to occur as frequently as three times per year.^7^

Yet quantifying the impact on human health per degree of global temperature change is difficult, and must take into account many confounding factors including behavior, says Christopher Portier, now director of the National Center for Environmental Health and the Agency for Toxic Substances and Disease Registry. In his former position as senior advisor at the National Institute of Environmental Health Sciences, Portier led a federal working group that released a report on 11 categories of disease and other health consequences that may occur due to climate change.^8^ That report highlighted a huge need for research to better understand the link between global warming and human health effects, Portier says.

The NRC report was released a few days before Senate majority leader Harry Reid (D–NV) announced there were not enough votes in support of climate change legislation, meaning Congress won’t pass a climate change bill in 2010. Tim Profeta, director of the Nicholas Institute for Environmental Policy Solutions at Duke University, says he read the NRC report the same day that climate legislation was failing in the Senate. “That created quite a juxtaposition,” he says, “showing us both the challenges we have before us and the amount of work that we have to get done.”

**Selected Potential Impacts^1^ (per degree global temperature increase)**

**1–2°C of Warming**

**FIRE**

- 200–400% increase in area burned per degrees in parts of western United States

**1–4°C of Warming**

**RAIN**

- 5–10% less rainfall per degree in Mediterranean, SW North America, southern Africa dry seasons
- 5–10% more rainfall per degree in Alaska and other high-latitude Northern Hemisphere areas
- 3–10% more heavy rain per degree in most land areas

**RIVERS**

- 5–10% less streamflow per degree in some river basins, including the Arkansas and Rio Grande

**FOOD**

- 5–15% reduced yield of U.S. corn, African corn, and Indian wheat per degree

**SEA ICE**

- 15% reduction in annual average Arctic sea ice area per degree

**3°C of Warming**

**COASTS**

- Loss of about 250,000 km^2^ of wet- and drylands
- Millions more people at risk of coastal flooding

**TEMPERATURE EXTREMES**

- 9 of 10 summer seasons are expected to be warmer than all but 1 summer of 20 in the last decades of the 20th century over nearly all land areas

**4°C of Warming**

**TEMPERATURE EXTREMES**

- About 9 out of 10 summers warmer than the warmest ever experienced during the last decades of the 20th century over nearly all land areas

**5°C of Warming**

**FOOD**

- Yield losses in most regions and potential doubling of global grain prices
